# Patterns of inpatient acute care and emergency department utilization within one year post-initial amputation among individuals with dysvascular major lower extremity amputation in Ontario, Canada: A population-based retrospective cohort study

**DOI:** 10.1371/journal.pone.0305381

**Published:** 2024-07-11

**Authors:** Sara J. T. Guilcher, Amanda L. Mayo, Sarah Swayze, Charles de Mestral, Ricardo Viana, Michael W. Payne, Steven Dilkas, Michael Devlin, Crystal MacKay, Ahmed Kayssi, Sander L. Hitzig

**Affiliations:** 1 Leslie Dan Faculty of Pharmacy, University of Toronto, Toronto, Ontario, Canada; 2 Institute of Health Policy Management and Evaluation, Dalla Lana School of Public Health, University of Toronto, Toronto, Ontario, Canada; 3 ICES, Toronto, Ontario, Canada; 4 Rehabilitation Sciences Institute, Temerty Faculty of Medicine, University of Toronto, Toronto, Ontario, Canada; 5 St. John’s Rehab Research Program, Sunnybrook Research Institute, Sunnybrook Health Sciences Centre, Toronto, Ontario, Canada; 6 Department of Physical Therapy, Temerty Faculty of Medicine, University of Toronto, Toronto, Ontario, Canada; 7 Division of Physical Medicine and Rehabilitation, Temerty Faculty of Medicine, University of Toronto, Toronto, Ontario, Canada; 8 Division of Vascular Surgery, Department of Surgery, Temerty Faculty of Medicine, University of Toronto, Toronto, Ontario, Canada; 9 Division of Vascular Surgery, St. Michael’s Hospital, Unity Health Toronto, Toronto, Ontario, Canada; 10 Li Ka Shing Knowledge Institute, St. Michael’s Hospital, Unity Health Toronto, Toronto, Ontario, Canada; 11 Department of Physical Medicine & Rehabilitation, Schulich School of Medicine & Dentistry, Western University, London, Ontario, Canada; 12 West Park Healthcare Centre, Toronto, Ontario, Canada; 13 Schulich Heart Research Program, Sunnybrook Research Institute, Sunnybrook Health Sciences Centre, Toronto, Ontario, Canada; 14 Department of Occupational Science & Occupational Medicine, Temerty Faculty of Medicine, University of Toronto, Toronto, Ontario, Canada; National Healthcare Group, SINGAPORE

## Abstract

**Introduction:**

Lower extremity amputation (LEA) is a life altering procedure, with significant negative impacts to patients, care partners, and the overall health system. There are gaps in knowledge with respect to patterns of healthcare utilization following LEA due to dysvascular etiology.

**Objective:**

To examine inpatient acute and emergency department (ED) healthcare utilization among an incident cohort of individuals with major dysvascular LEA 1 year post-initial amputation; and to identify factors associated with acute care readmissions and ED visits.

**Design:**

Retrospective cohort study using population-level administrative data.

**Setting:**

Ontario, Canada.

**Population:**

Adults individuals (18 years or older) with a major dysvascular LEA between April 1, 2004 and March 31, 2018.

**Interventions:**

Not applicable.

**Main outcome measures:**

Acute care hospitalizations and ED visits within one year post-initial discharge.

**Results:**

A total of 10,905 individuals with major dysvascular LEA were identified (67.7% male). There were 14,363 acute hospitalizations and 19,660 ED visits within one year post-discharge from initial amputation acute stay. The highest common risk factors across all the models included age of 65 years or older (versus less than 65 years), high comorbidity (versus low), and low and moderate continuity of care (versus high). Sex differences were identified for risk factors for hospitalizations, with differences in the types of comorbidities increasing risk and geographical setting.

**Conclusion:**

Persons with LEA were generally more at risk for acute hospitalizations and ED visits if higher comorbidity and lower continuity of care. Clinical care efforts might focus on improving transitions from the acute setting such as coordinated and integrated care for sub-populations with LEA who are more at risk.

## Introduction

Lower extremity amputation (LEA) is a life altering procedure, with significant negative impacts to patients, caregivers, and the overall health system [1–5]. Worldwide, LEAs are a leading cause of disability [6]. The annual incidence of LEA is estimated to widely range from 8.8–92.5 per 100,000 persons [7], with higher risks among more equity-seeking populations such as those with lower socioeconomic status and certain ethnicities (e.g., Black and Hispanic populations) [8, 9]. There are two main types of LEA, major and minor. Major LEA includes transfemoral (above-knee), knee disarticulation (through-knee), trans-tibial (below-knee), and Syme (through-ankle); while minor LEA relate to partial foot and toe amputations and are generally performed in an effort to avoid major LEA. Main etiologies for LEA include traumatic related injuries, cancer, congenital limb deformities, with over 80% due to dysvascular disease (e.g., complications from diabetes and/or vascular diseases) [7, 10]. Unfortunately, the incidence of LEA is expected to rise [10, 11], mostly due to rising prevalence of diabetes [12].

Long term clinical outcomes among persons with dysvascular LEA are relatively poor, with high morbidity and mortality rates following amputation [13–15]. Diabetes-related LEA is one of the leading causes of the global burden of disability [15]. Throud and colleagues identified in their systematic review that the overall five year mortality rate ranged from 53% to 80% among persons with major LEA due to diabetes and peripheral vascular disease [13]. Given these sub-optimal outcomes, it is important to examine patterns of healthcare utilization following a LEA to inform service access, delivery, care management and experiences [16]. Furthermore, understanding who might be more at risk within the LEA population, such as those with dysvascular etiologies [17, 18], may inform tailored and targeted supports to assist with prevention and management of readmission and emergency department (ED) visits after LEA. For example, social determinants of health play a key role in the risk factors, prevention, and management of LEA [19]. Recent research in the United States has shown that persons identifying as Black and living in rural settings are particularly at risk of a first dysvascular LEA [19, 20] compared to those who were non-Black living in urban settings [20]. In Canada, recent research has shown Indigenous persons are at increased risk for subsequent LEA [21].

Currently, there is limited research in Canada on patterns of healthcare utilization following dysvascular LEA [22]. Using population health data, the purpose of this study was to examine patterns of acute healthcare utilization (acute care visits and emergency department [ED] visits) within the one year following an initial major dysvascular LEA and associated risk factors for healthcare utilization.

## Methods

### Study design and setting

We used linked administrative healthcare data from ICES (formerly known as the Institute for Clinical Evaluative Sciences) to conduct a retrospective study of persons in Ontario, Canada with major dysvascular LEA. As a province in Canada that follows the Canada Health Act, Ontario has a universal health insurance program for its almost 15 million residents, which covers hospitalization and other medically necessary care including inpatient rehabilitation, complex continuing care and physician visits. ICES (www.ices.on.ca) is an independent, non-profit research institute funded by an annual grant from the Ontario Ministry of Health and the Ontario Ministry of Long-Term Care. As a prescribed entity under Ontario’s privacy legislation, ICES is authorized to collect and use healthcare data for the purposes of health system analysis, evaluation and decision support. Secure access to these data is governed by policies and procedures that are approved by the Information and Privacy Commissioner of Ontario. The use of data was authorized under section 45 of Ontario’s *Personal Health Information Protection Act*, which does not require review by a Research Ethics Board. However, we acquired ethics through Sunnybrook Research Institute (# 2588) and the University of Toronto (# 36086).

### Databases

We used several administrative healthcare datasets housed at ICES. These datasets were linked using unique encoded identifiers and analyzed at ICES to capture the delivery of healthcare services in Ontario. Numerous studies have assessed and verified the validity of these databases [23–25]. We used the Canadian Institute for Health Information (CIHI)’s Discharge Abstract Database to capture records of all hospitalizations and diagnoses/procedures that occur in hospital. The Ontario Mental Health Reporting System (OMHRS) was used to capture mental health admissions. We obtained diagnostic information on ED visits, same day surgeries and specialty ambulatory clinic visits using the National Ambulatory Care Reporting System. We captured physician visits, including diagnostic codes, physician specialty and location of the service using the Ontario Health Insurance Program database. The National Rehabilitation Reporting System provided admission and discharge dates for inpatient rehabilitation care. We used the Ontario Registered Persons database to obtain demographic information (e.g., sex, age, date of birth, residential postal code). We used the Ontario Drug Benefit Database to capture whether prescription medications were dispensed to individuals insured through the provincial drug plan in the year prior to the initial amputation admission. The provincial drug plan covers persons for drug coverage if they are 65 years of age or older, living in long-term care, receive home care services, have high prescription medication costs compared to their net household income, or receive social financial assistance (e.g., Ontario Works or Ontario Disability Support Program).

### Study cohort

**Fig 1:**
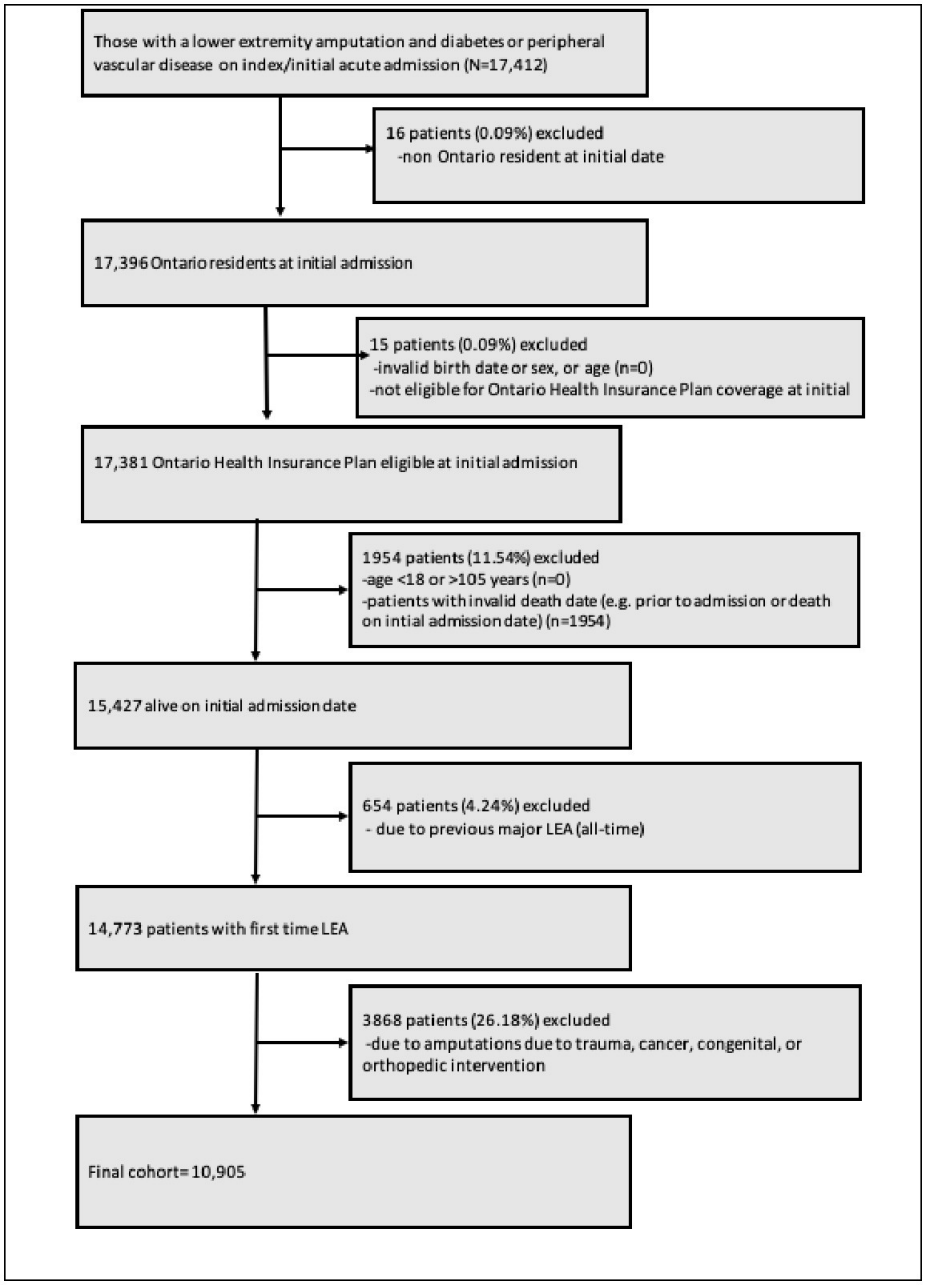
Cohort flow chart (N = 10,905). Cohort flow chart provides an overview of eligibility criteria for study.

We included individuals with an initial major dysvascular LEA between April 1, 2004 and March 31, 2018. The following Canadian Classification of Health Intervention (CCI) codes were used to identify major LEA: 1VC93, 1VG93, 1VQ93, and 1WA93. Persons also needed to have a diagnosis of peripheral vascular disease and/or diabetes prior to or at the time of initial LEA hospitalization [26, 27]. We excluded those not eligible for health coverage in Ontario or a non-resident of Ontario, invalid death date (died before admission, or died during initial stay), those who were less than 18 years of age or older than 105 years, missing or invalid sex or date of birth, those with a previous major dysvascular LEA event (looked back to 2002), and those with amputations due to trauma, cancer, congenital, or orthopedic causes (see Fig 1 for cohort flow diagram).

### Variables of interest

Sociodemographic characteristics included sex, age, rurality, neighbourhood level income, and neighbourhood racialized and newcomer population (formerly called ethnic concentration) on the date of admission for the major dysvascular amputation. Clinical characteristics included level of amputation (above/through knee, and below knee), previously diagnosed comorbidities (coronary syndrome, osteoarthritis, rheumatoid arthritis, asthma, cancer, heart arrythmias, congestive heart failure, chronic obstructive pulmonary disease, diabetes, dementia, hypertension, osteoporosis, renal disease, stroke and mental health conditions [non-psychotic mood and anxiety disorders and other mental health conditions]), previous prescription drug dispensed (for those individuals eligible for Ontario Drug Benefit Plan) [28–35]. We used the Johns Hopkins ACG^®^ System (v10) to calculate Aggregated Diagnosis Groups (ADGs), which were used to describe morbidity burden. This proprietary software uses hospitalization, ED, and physician office visits during the two years prior to the observation period and a higher ADG score represents higher morbidity burden. We also identified the number of chronic comorbidities using a multimorbidity macro which leverages several ICES-derived cohorts from various datasets, including the Ontario Asthma Dataset, Congestive Heart Failure Dataset, Chronic Obstructive Pulmonary Disease Dataset, Ontario Hypertension Dataset, Ontario Diabetes Dataset, Ontario Rheumatoid Arthritis Dataset, and the Ontario Dementia Database. Continuity of Care was calculated using the Ambulatory Usual Provider of Care index (0–100), which determines the proportion of all ambulatory care visits (most frequent billing physician) in the one year following discharge from acute care to the most highly visited provider. A threshold of 75% of visits with the same physician indicates high continuity of care [36, 37]. Neighbourhood level income was determined by postal code information in the Registered Persons Database. Racialized and newcomer population (previously called ethnic concentration) was identified using the Ontario Marginalization Index [38]. Urban and rural residential setting were determined using the Rurality Index for Ontario (RIO) [39]. The RIO uses census data to calculate a score, with 0–39 indicating an urban area and 40 or greater as rural [39].

#### Outcomes

Main outcomes included the number of acute care hospitalizations and ED visits during the one year period post discharge from initial amputation acute care stay. We captured the main reasons for admission using the International Classification of Diseases Canada Version (ICD-10 CA), with the main diagnosis codes.

### Statistical analysis

We used descriptive statistics to characterize our study cohort (e.g., mean, medians, standard deviations (SD) and interquartile range (IQR)). For differences between males and females, we used chi-square tests for categorical variables and t-tests for continuous variables. We used negative binomial regression models to characterize variables associated with acute hospitalizations and ED use within one year post discharge from acute initial amputation stay. We included the following variables in the models: sex (male, female), age (65+, <65), ADG score (low [0–5], moderate [6–9], high [10+]), inpatient rehabilitation post amputation (yes, no), level of amputation (above/through, below knee), income quintile (low [1–3 quintile], high [4–5 quintile]), any diagnosis of cardiovascular and cerebral vascular disease (yes, no), chronic obstructive pulmonary disease (yes, no), or mental health condition (yes, no), continuity of care (low [<50%], moderate [50-<75%], high [75%+]), rurality [rural, urban], and racialized and newcomer quintiles (least racialized and newcomer concentrated quintiles [1,2,3], most racialized and newcomer concentrated quintiles [4,5]). The final variables in the adjusted models were determined based on expert clinical opinion and examination of univariate models. The adjusted model for acute care hospitalizations was stratified by sex due to possible differences in physiology, lifestyle, and post-amputation quality of life [7, 10, 40–42]. We stratified ED models by rural and urban due to known patterns of visits varying by geography in the general population [43–45]. Multicollinearity was checked within models. We calculated adjusted relative risks (aRR) with 95% confidence intervals. Statistical significance was set at P ≤ 0.05. All data were analyzed with SAS version 9.4 (SAS Institute Inc., Cary, NC; www.sas.com) at ICES.

## Results

### Characteristics of the cohort

A total of 10,905 persons with major dysvascular LEA were included in the study (Table 1). The mean age was 68.9 (SD = 12.9), and the majority were male (n = 7,384, 67.7%). Most individuals had a below knee amputation (65.9%), and just under a third of the cohort went to inpatient rehabilitation within the year following the amputation (30.2% n = 3,569). Most individuals were living in the lower neighborhood income quintiles (71.5% in income quintiles 1–3) and in urban areas (89.0%). The median number of ADGs in the cohort was 11 (IQR = 8–14), with most of the cohort having high comorbidity of ADG 10+ (n = 6,974, 64.0%). Almost a third of the cohort had five or more chronic conditions (n = 3,424, 31.4%). The most common chronic conditions included diabetes (76.3%), hypertension (69.8%), osteoarthritis (35.0%), any mental health condition (28.3%), renal failure (29.3%), coronary syndrome (29.5%), chronic heart failure (28.5%), and chronic obstructive pulmonary disease (16.6%). The median number of drug classes dispensed in the previous year was 15 (IQR = 11–19).

**Table 1:** Characteristics of individuals with a major lower limb amputation due to dysvascular condition in Ontario, Canada (fiscal years 2004–2018).

Variable	Female	Male	Total	P value*
	N = 3,521	N = 7,384	N = 10,905	
**Age (years)**				
Mean ± SD	71.2 ± 13.7	67.9 ± 12.4	68.9 ± 12.9	<0.001
Median (IQR)	73 (61–82)	68 (60–77)	69 (60–79)	<0.001
**Senior (65+ years), n (%)**				
No	1,198 (34.0)	3,098 (42.0)	4,296 (39.4)	<0.001
Yes	2,323 (66.0)	4,286 (58.0)	6,609 (60.6)	
**Amputation level, n (%)**				
Below knee	2,035 (57.8)	5,152 (69.8)	7,187 (65.9)	<0.001
Above/through knee	1,486 (42.2)	2,232 (30.2)	3,718 (34.1)	
**Inpatient rehab post-initial event, n (%)**				
*Within one year post-initial*				
No	2,634 (74.8)	4,976 (67.4)	7,610 (69.8)	<0.001
Yes	887(25.2)	2,408 (32.6)	3,569 (30.2)	
**Rural index, n (%)**				
Urban	3,174 (90.1)	6,526 (88.4)	9,700 (89.0)	0.006
Rural	347 (9.9)	858 (11.6)	1,205 (11.0)	
**Neighbourhood income, n (%)**				
1–3 quintiles (low)	2,507 (71.2)	5,289 (71.6)	7,796 (71.5)	0.645
4–5 quintiles (high)	1,014 (28.8)	2,095 (28.4)	3,109 (28.5)	
**Racialized and newcomer population, n (%)**				
1–3 quintiles least concentrated	2,206 (62.7)	4,781 (64.7)	6,987 (64.1)	0.033
4–5 quintiles most concentrated	1,315 (37.3)	2,603 (35.3)	3,918 (35.9)	
**Comorbidity (ADG)**,^†^ **n (%)**				
Low (0–5)	345 (9.8)	839 (11.4)	1,184 (10.9)	<0.001
Moderate (6–9)	827 (23.5)	1,920 (26.0)	2,747 (25.2)	
High (10+)	2,349 (66.7)	4,625 (62.6)	6,974 (64.0)	
**Number of comorbidities, n (%)**				
0	46 (1.3)	102 (1.4)	148 (1.4%)	0.008
1	299 (8.5)	724 (9.8)	1,023 (9.4%)	
2	580 (16.5)	1,336 (18.1)	1,916 (17.6)	
3	742 (21.1)	1,584 (21.5)	2,326 (21.3)	
4	722 (20.5)	1,346 (18.2)	2,068 (19.0)	
5+	1,132 (32.1)	2,292 (31.0)	3,424 (31.4)	
**Diagnosis of COPD, n (%)**	593 (16.8)	1,216 (16.5)	1,809 (16.6)	0.624
**Diagnosis of cancer, n (%)**	627 (17.8)	1,350 (18.3)	1,977 (18.1)	0.547
**Diagnosis of diabetes, n (%)**	2,453 (69.7)	5,865 (79.4)	8,318 (76.3)	<0.001
**Diagnosis of renal failure, n (%)**	978 (27.8)	2,222 (30.1)	3,200 (29.3)	0.013
**Diagnosis of osteoarthritis, n (%)**	1,380 (39.2)	2,435 (33.0)	3,815 (35.0)	<0.001
**Diagnosis of acute myocardial infarction, n (%)**	164 (4.7)	375 (5.1)	539 (4.9)	0.343
**Diagnosis of coronary syndrome, n (%)**	944 (26.8)	2,270 (30.7)	3,214 (29.5)	<0.001
**Diagnosis of chronic heart failure, n (%)**	1,049 (29.8)	2,062 (27.9)	3,111 (28.5)	0.043
**Diagnosis of hypertension, n (%)**	2,564 (72.8)	5,048 (68.4)	7,612 (69.8)	<0.001
**Diagnosis of stroke, n (%)**	242 (6.9)	494 (6.7)	736 (6.7)	0.722
**Diagnosis of any cardiovascular, cerebrovascular conditions, n (%**)^††^	2,915 (82.8)	5,890 (79.8)	8,805 (80.7)	<0.001
**Diagnosis of osteoporosis, n (%)**	88 (2.5)	47 (0.6)	135 (1.2)	<0.001
**Diagnosis of rheumatoid arthritis, n (%)**	126 (3.6)	96 (1.3)	222 (2.0)	<0.001
**Diagnosis of dementia, n (%)**	194 (5.5)	255 (3.5)	449 (4.1)	<0.001
**Diagnosis of mood, anxiety, depression and non-psychotic mood disorders, n (%)**	871 (24.7)	1,383 (18.7)	2,254 (20.7)	<0.001
**Diagnosis of other mental illnesses, n (%)**	351 (10.0)	1,003 (13.6)	1,354 (12.4)	<0.001
**Diagnosis of any mental health conditions, n (%)**	1,057 (30.0)	2,025 (27.4)	3,082 (28.3)	0.005
**Usual Provider of Care Index**				
No health system contact <3 visits	83 (2.4)	121 (1.6)	204 (1.9)	<0.001
Low (<0.50)	1,753 (49.8)	4,362 (59.1)	6,115 (56.1)	
Moderate (0.50-<0.75)	884 (25.1)	1,716 (23.2)	2,600 (23.8)	
High (≥0.75)	801 (22.7)	1,185 (16.0)	1,986 (18.2)	

*p value is comparing males and females using chi-square tests for categorical variables and t-tests for continuous variables

^†^ Lookback for ADG comorbidity included two years prior to the initial amputation

^††^ Includes acute myocardial infarction, congestive heart failure, coronary syndrome, and cerebral vascular disease

ED = Emergency department; ADG = Aggregated Diagnosis Group; COPD = Chronic Obstructive Pulmonary Disease

### Acute hospitalizations within one year post initial amputation

There were 14,363 hospitalizations (60.4% of individuals with at least 1 acute care hospitalization) within one year post discharge (Table 2). The median total length of stay was 14 days (IQR = 6–32), acute care was 12 days (IQR = 5–25) and delay in discharge (known as alternate level of care in Canada) was 10 days (IQR = 4–30). The top reasons for admission within one year included the following ICD-10 chapters (Table 3): “Injury, poisoning and other external causes (S00-T98)” (19.3%), “Diseases of the circulatory system (I00-I99)” (19.1%), and “Endocrine, nutritional, and metabolic diseases (E00-E90)” (16.7%). The top most responsible diagnostic codes were the following: congestive heart failure (I50.0; n = 533, 5.1%); septicemia (A41.9; n = 404, 3.8%); encounter for palliative care (Z51.5; n = 356; 3.4%); complications to reattachment and amputation (T87.47; n = 311, 3.0%); and type 2 diabetes mellitus with peripheral circulatory complications (E11.51; n = 300, 2.9%; see S1 Table).

**Table 2:** Characteristics of hospitalizations and emergency department visits among persons with major lower limb amputation due to dysvascular condition in Ontario, Canada (fiscal years 2004–2018).

Variable	Female	Male	Total	P value*
	N = 3,521	N = 7,384	N = 10,905	
**Total number of acute hospitalizations in one year following amputation**	4,468	9,895	14,363	
**Individuals with at least 1 acute hospitalization in one year following amputation, n (%)**	2,103 (59.7)	4,485 (60.7)	6,588 (60.4)	0.31
**Acute hospitalization in one year following amputation, n (%)**				
0	1,418 (40.3)	2,899 (39.3)	4,317 (39.6)	0.44
1	961 (27.3)	1,992 (27.0)	2,953 (27.1)	
2	557 (15.8)	1,144 (15.5)	1,701 (15.6)	
3	274 (7.8)	631 (8.5)	905 (8.3)	
4	152 (4.3)	338 (4.6)	490 (4.5)	
5+	159 (4.5)	380 (5.1)	539 (4.9)	
**Length of stay for acute hospitalizations**				
Total days median (IQR)	14 (5–31)	14 (6–33)	14 (6–32)	0.36
Acute days median (IQR)	13 (5–25)	12 (5–25)	12 (5–25)	0.14
Delayed discharge days median (IQR)^†^	9 (3–24)	11 (5–30)	10 (4–30)	0.12
**Total number of ED visits in one year following amputation**	6,162	13,498	19,660	
**Individuals with at least 1 ED visit in one year following amputation, n (%)**	2,191 (62.2)	4,591 (62.2)	6,782 (62.2)	0.96
**ED visit in one year following amputation, n (%)**				
0	1,330 (37.8)	2,793 (37.8)	4,123 (37.8)	0.35
1	847 (24.1)	1,726 (23.4)	2,573 (23.6)	
2	491 (13.9)	1,108 (15.0)	1,599 (14.7)	
3	313 (8.9)	624 (8.5)	937 (8.6)	
4	201 (5.7)	375 (5.1)	576 (5.3)	
5+	339 (9.6)	758 (10.3)	1,097 (10.1)	
**ED visit associated with an acute admission**				
No	2,041 (33.1)	4,536 (33.6)	6,577 (33.5)	0.51
Yes	4,121 (66.9)	8,962 (66.4)	13,083 (66.5)	
**Died within one year after initial amputation**				
No	2,595 (73.7)	5,804 (78.6)	8,399 (77.0)	<0.001
Yes	926 (26.3)	1,580 (21.4)	2,506 (23.0)	

*p value is comparing males and females using chi-square tests for categorical variables and t-tests for continuous variables

^†^ Among persons with a delay in discharge > 0 days

ED = Emergency department

**Table 3:** Top 15 reasons for acute care admissions within one year post-initial amputation based on the International-Classification of Diseases (ICD) 10th version.

Reason for Visit by Chapter	n (%)
Injury, poisoning and certain other consequences of external causes (S00-T98)	2030 (19.3)
Diseases of the circulatory system (I00-I99)	2005 (19.1)
Endocrine, nutritional and metabolic diseases (E00-E90)	1761 (16.7)
Diseases of the respiratory system (J00-J99)	754 (7.2)
Certain infectious and parasitic diseases (A00-B99)	725 (6.9)
Diseases of the digestive system (K00-K93)	614 (5.8)
Diseases of the genitourinary system (N00-N99)	609 (5.8)
Symptoms, signs and abnormal clinical and laboratory findings, not elsewhere classified (R00-R99)	573 (5.5)
Factors influencing health status and contact with health services (Z00-Z99)	503 (5.5)
Diseases of the musculoskeletal system and connective tissue (M00-M99)	226 (4.8)
Diseases of the skin and subcutaneous tissue (L00-L99)	223 (2.2)
Neoplasms (C00-D48)	187 (1.8)
Mental and behavioural disorders (F00-F99)	121 (1.2)
Diseases of the blood and blood-forming organs and certain disorders involving the immune mechanism (D50-D89)	92 (0.8)
Diseases of the nervous system (G00-G99)	81 (0.8)

### ED visits within one year post initial amputation

Most of the cohort had an ED visit within one year (62.2%), with 19,660 visits, and 65.5% of visits led to an acute hospitalization (n = 13,083; Table 2). The median number of visits was 3 (IQR = 1–5). Main reasons for ED visits within one year included the following ICD-10 chapters (Table 4): “Symptoms, signs and abnormal clinical and laboratory findings, not elsewhere classified” (R00-R99, 16.8%), “Injury, poisoning and other external causes” (S00-T98; 15.7%), and “Factors influencing health status and contact with health services “(Z00-Z99, 11.8%). More specific top most responsible diagnostic codes were: Urinary tract infection site not specified (N39.0; n = 676; 3.4%); chemotherapy (Z51.2; n = 601; 3.1%), congestive heart failure (I50.0; n = 591; 3.0%); cellulitis (L03.11; n = 581; 3.0%); and pneumonia not otherwise specified (J18.9; n = 468; 2.4%; S1 Table).

**Table 4:** Top 15 reasons for emergency department visits within one year post-initial amputation based on the International-Classification of Diseases (ICD) 10th version.

Reason for Visit by Chapter	n (%)
Symptoms, signs and abnormal clinical and laboratory findings, not elsewhere classified (R00-R99)	3302 (16.8)
Injury, poisoning and certain other consequences of external causes (S00-T98)	3092 (15.7)
Factors influencing health status and contact with health services (Z00-Z99)	2322 (11.8)
Diseases of the circulatory system (I00-I99)	2048 (10.4)
Endocrine, nutritional and metabolic diseases (E00-E90)	1877 (9.6)
Diseases of the skin and subcutaneous tissue (L00-L99)	1221 (6.21)
Diseases of the respiratory system (J00-J99)	1157 (5.9)
Diseases of the genitourinary system (N00-N99)	1052 (5.4)
Diseases of the digestive system (K00-K93)	929 (4.7)
Certain infectious and parasitic diseases (A00-B99)	840 (4.3)
Diseases of the musculoskeletal system and connective tissue (M00-M99)	676 (3.4)
Mental and behavioural disorders (F00-F99)	358 (1.8)
Diseases of the blood and blood-forming organs and certain disorders involving the immune mechanism (D50-D89)	277 (1.4)
Diseases of the nervous system (G00-G99)	244 (1.2)
Diseases of the eye and adnexa (H00-H59)	129 (0.7)

### Predictors of hospitalizations within one year

Sex differences were identified in examining the data for hospitalizations, and consequently the models were stratified by sex (Table 5). Risk factors for acute hospitalization within one year for females included the following: being 65+ years of age compared to less than 65 years (aRR = 1.37; 95% CI 1.26–1.48), high (aRR = 1.61; 95% CI 1.39–1.89) and moderate (aRR = 1.20; 95% CI 1.02–1.42) comorbidity compared with low comorbidity, having cardiovascular or cerebrovascular conditions compared with none (aRR = 1.13; 95% CI 1.01–1.26), having chronic obstructive pulmonary disease compared with none (aRR = 1.30; 95% CI 1.18–1.44), having a mental health condition compared with none (aRR = 1.10; 95% CI 1.01–1.20), low continuity of care compared to high continuity of care (aRR = 1.83; 95% CI 1.63–2.05), moderate continuity of care compared to high continuity of care (aRR = 1.47; 95% CI 1.30–1.66), and living in a rural setting compared to urban setting (aRR = 1.16; 95% CI 1.02–1.32).

**Table 5:** Risk factors for acute care hospitalizations within one year for individuals with initial dysvascular major amputations between fiscal years 2004–2018, stratified by sex, in Ontario, Canada.

Variable	Females		Males	
	Adjusted Relative Risk	P value	Adjusted Relative Risk	P value
Seniors (65 years+) Yes (reference No)	1.37 (1.26–1.48)	<0.001	0.99 (0.93–1.05)	0.70
ADG^†^ High 10+ (reference low)	1.61 (1.39–1.89)	<0.001	1.68 (1.52–1.86)	<0.001
ADG Moderate 6–9 (reference low)	1.20 (1.02–1.42)	0.03	1.13 (1.01–1.26)	0.03
Low income (1,2,3) (reference high (4,5))	1.02 (0.93–1.11)	0.66	1.04 (0.97–1.10)	0.26
Amputation above/through (reference below)	0.95 (0.88–1.03)	0.24	0.93 (0.87–0.99)	0.02
No inpatient rehabilitation (reference yes)	1.05 (0.96–1.15)	0.30	1.04 (0.98–1.10)	0.18
Cardiovascular/Cerebrovascular condition^††^ (reference no)	1.13 (1.01–1.26)	0.03	1.21 (1.12–1.30)	<0.001
Chronic obstructive pulmonary disease (reference no)	1.30 (1.18–1.44)	<0.001	1.18 (1.10–1.27)	<0.001
Mental health condition (reference no)	1.10 (1.01–1.20)	0.03	0.99 (0.93–1.05)	0.67
Usual Provider of Care Index Low (<0.50) (reference high)	1.83 (1.63–2.05)	<0.001	1.82 (1.67–1.99)	<0.001
Usual Provider of Care Index Moderate (0.50-<0.75) (reference high)	1.47 (1.30–1.66)	<0.001	1.49 (1.35–1.64)	<0.001
Neighbourhood racialized and newcomer population most (reference least)	1.03 (0.95–1.12)	0.42	0.99 (0.94–1.05)	0.85
Rural setting (reference urban)	1.16 (1.02–1.32)	0.03	1.03 (0.94–1.12)	0.56

^†^ Lookback for ADG comorbidity included two years prior to the initial amputation

^††^ Includes acute myocardial infarction, congestive heart failure, coronary syndrome, and cerebral vascular disease

ADG = Aggregated Diagnosis Group; COPD = Chronic Obstructive Pulmonary Disease

Risk factors for acute hospitalization within one year for males included high comorbidity compared with low comorbidity (aRR = 1.68; 95% CI 1.52–1.86), moderate comorbidity compared with low comorbidity (aRR = 1.13; 95% CI 1.01–1.26), having cardiovascular/cerebrovascular conditions compared with none (aRR = 1.21; 95% CI 1.12–1.30), having chronic obstructive pulmonary disease compared with none (aRR = 1.18; 95% CI 1.10–1.27), and low continuity of care compared with high (aRR = 1.82; 95% CI 1.67–1.99), and moderate continuity of care compared to high continuity of care (aRR = 1.49; 95% CI 1.35–1.64). An amputation above/through the knee was protective compared to below the knee (aRR = 0.93; 95% CI 0.87–0.99).

### Predictors of ED within one year

Risk factors for ED visits within one year for those living in urban areas included being 65 years of age or older compared to those less than 65 years (aRR = 1.30; 95%CI 1.23–1.37), high (aRR = 1.92; 95% CI 1.74–2.11) and moderate (aRR = 1.39; 95% CI 1.25–1.54) comorbidity compared to low, living in a neighbourhood of lower income (aRR = 1.13; 95%CI 1.06–1.20), diagnosis of chronic obstructive pulmonary disease (aRR = 1.29; 95% CI 1.20–1.38), having any mental health condition (aRR = 1.10; 95% CI 1.04–1.17), and low (aRR = 1.68; 95% CI 1.56–1.81) and moderate (aRR = 1.45; 95% CI 1.33–1.58) continuity of care compared to high (Table 6). Patients with an amputation above/through the knee was protective compared to below the knee (aRR = 0.93; 95%CI 0.88–0.99).

**Table 6:** Risk factors for emergency department visits within one year for individuals with initial dysvascular major amputations between fiscal years 2004–2018, in Ontario, Canada, stratified by geography.

Variable	Urban		Rural	
	Adjusted Relative Risk	P value	Adjusted Relative Risk	P value
Male (Reference female)	1.02 (0.97–1.08)	0.48	0.88 (0.74–1.04)	0.14
Seniors (65 years+) Yes (reference No)	1.30 (1.23–1.37)	<0.001	1.42 (1.20–1.68)	<0.001
ADG High 10+ (reference low)	1.92 (1.74–2.11)	<0.001	1.43 (1.07–1.91)	0.01
ADG Moderate 6–9 (reference low)	1.39 (1.25–1.54)	<0.001	0.92 (0.68–1.25)	0.61
Low income (1,2,3) (Reference high (4,5))	1.13 (1.06–1.20)	<0.001	1.19 (1.00–1.40)	0.04
Amputation above/through knee (reference below)	0.93 (0.88–0.99)	0.02	0.98 (0.82–1.17)	0.83
No Inpatient rehabilitation (reference yes)	1.09 (0.97–1.23)	0.14	0.92 (0.62–1.38)	0.69
Cardiovascular/Cerebrovascular condition (reference no)	1.05 (0.98–1.13)	0.18	1.01 (0.84–1.22)	0.89
Chronic obstructive pulmonary disease (reference no)	1.29 (1.20–1.38)	<0.001	1.28 (1.05–1.58)	0.02
Mental health condition (reference no)	1.10 (1.04–1.17)	<0.001	1.11 (0.92–1.34)	0.27
Usual Provider of Care Index Low (reference high)	1.68 (1.56–1.81)	<0.001	1.72 (1.38–2.13)	<0.001
Usual Provider of Care Index Moderate (reference high)	1.45 (1.33–1.58)	<0.001	1.59 (1.24–2.03)	<0.001
Neighbourhood racialized and newcomer population most (reference least)	1.02 (0.97–1.08)	0.38	0.86 (0.51–1.44)	0.56

^†^ Reference refers to the category used as a comparator in the model

Abbreviations ADGs = Aggregated Diagnosis Groups

Risk factors for ED visits within one year for those living in rural areas included being 65 years of age or older compared to those less than 65 years (aRR = 1.42; 95%CI 1.20–1.68), high comorbidity compared to low (aRR = 1.43; 95% CI 1.07–1.91), living in area with lower income (aRR = 1.19; 95%CI 1.00–1.40), diagnosis of chronic obstructive pulmonary disease (aRR = 1.28; 95% CI 1.05–1.58), and low (aRR = 1.72; 95% CI 1.38–2.13) and moderate (aRR = 1.59; 95% CI 1.24–2.03) continuity of care compared with high (Table 6).

## Discussion

In this population-based administrative health data study, we characterized acute care hospitalizations and ED visits within one year following amputation among persons with major dysvascular amputations. Overall, we found that 62.2% of persons visited the ED, and 60.4% were re-admitted to acute care within the year. The most common reasons for acute re-admissions and ED visits related to infections (urinary tract, pulmonary and sepsis), congestive heart failure/myocardial infarctions, diabetes, chronic obstructive pulmonary disease, and wound-related care. Common risk factors for rehospitalization included having lower continuity of care, increasing comorbidity, and having a diagnosis of chronic obstructive pulmonary disease. However, sex differences were also identified, with females being at risk for re-hospitalization if a previous diagnosis of a mental health condition. Persons with LEA were more at risk for ED visits if they also had higher comorbidity, lower continuity of care, and living in areas of lower income. These findings suggest that clinical care efforts might focus on integrated care, particularly within primary and specialist care, to assist in the management of LEA and related conditions.

Despite substantial changes over the past twenty years in Canada with a focus on primary care reform [46], the high rates of rehospitalization for persons with dysvascular LEA seem to be relatively unchanged. For example, Kayssi and colleagues identified that 55.4% of persons with LEA (2006 to 2008) in Canada were readmitted within the year following a LEA [47]. In the present study, persons were more at risk if they had substantial medical and social complexity (e.g., higher comorbidity). Similar to Kayssi [47], we also identified sex differences, with females at increased risk for re-hospitalization if previously diagnosed with a mental health condition, increasing comorbidity, previously diagnosed with cardiovascular/cerebrovascular disease, previously diagnosed with chronic obstructive pulmonary disease and living in a rural setting.

Our findings of previous diagnosis of mental health conditions as a risk factor for ED visits and rehospitalization among females may assist in flagging certain sub-groups who may be more in need of support. Previous research has shown prevalence rates for depression ranging from 20% to 63% and for anxiety 25% to 57% among persons following their LEA [48, 49], which is significantly higher than the general population [49]. In our study, we did not examine the rates of mental health diagnoses following a LEA but rather the diagnoses prior to the amputation, which would be an important area of study using population health data. Minimal research exists examining the change over time of mental health among person with LEA but there is evidence highlighting that this is a population with several unmet needs with regards to their mental health [50].

Importantly, our study identified improved continuity of care was protective for ED visits as well as re-hospitalizations within the one year period following initial acute LEA. These findings are consistent with previous work, that has shown better continuity of care is associated with quality primary care and overall better patient outcomes [51–58]. Relatedly, health jurisdictions are shifting from solo care models to integrated medical homes to improve continuity of care [59–61]. Our data suggests that persons with LEA need more comprehensive care (including mental health support) following their amputation to optimize community re-integration. Patient navigator models have been shown to be effective with supporting other patient populations groups, such as those with chronic kidney disease [62], and cancer-related services [63].

Given the complex medical profile of persons with LEA, a focus on self-management support within a team-based model of care, may assist with overall physical, emotional, and social functioning of persons with LEA [64]. Self-management refers to the tasks, skills and behaviours associated with an individual’s ability to navigate the physical, social, and cognitive lifestyle factors, changes, and consequences of living with a chronic condition [65]. Fostering self-management skills have been shown to assist with improved care transitions [66, 67] and ability to function with chronic conditions in everyday life [68–70]. Previous qualitative research identified the need for more self-management education and training opportunities within rehabilitation to assist in the transition from hospital to community for persons with LEA [71]. To date, there is limited research on self-management and LEA [64]. In a recent systematic review, only 7 articles were identified on self-management among persons with LEA [64]. However, it is conceivable that the core skills required in other populations with chronic conditions would apply to persons with LEA, such as problem-solving, decision-making, seeking formal and informal supports, self-tailoring, goal-setting, optimizing social interactions, and engaging in activities, as they relate to everyday life [65]. The opportunity to learn and foster self-management skills might assist individuals coping with the LEA and their other comorbid conditions, as well as transitioning to the community following surgery.

### Limitations

This study has a few limitations that need to be acknowledged. The data reflect healthcare use patterns prior to the COVID-19 pandemic, and as such, may not reflect healthcare delivery and use after onset of the pandemic to present day. However, challenges with access to care and care management likely still exist. Further research is warranted to explore the impact of COVID-19 and post-pandemic on access to services and whether care needs have been met for this population. The administrative data are limited in potentially important social determinant variables (e.g., ethnicity, gender, functional abilities). Drug information was limited to those eligible for the Ontario Drug Benefit Plan (e.g., 65 years or older, receiving social assistance for low income or disability). More extensive exploration of mental health needs and supports provided for persons with LEA would also be useful, particularly understanding unique needs of certain sub-populations such as those with lower income, increasing comorbidity, and sex/gender considerations.

## Conclusions

Persons with dysvascular major LEA have significant comorbidity and healthcare utilization following the amputation. In general, persons were more at risk for hospitalizations and ED visits if higher comorbidity and lower continuity of care. Clinical care efforts might focus on improving integrated care and self-management support for sub-populations with LEA who are more at risk.

## Supporting information

S1 Table Top 10 reasons for admission by International-Classification of Diseases (ICD) 10th version codes. Table summarizing the top reasons for admission to inpatient acute care and the emergency department.
(DOCX)
