# Supplementary material for: Patterns of inpatient acute care and emergency department utilization within one year post-initial amputation among individuals with dysvascular major lower extremity amputation in Ontario, Canada: A population-based retrospective cohort study
Source: PLoS One. 2024 Jul 11;19(7):e0305381. doi: 10.1371/journal.pone.0305381 (PMC11238985; doi:10.1371/journal.pone.0305381)
Supplement: S1 Table — Table summarizing the top reasons for admission to inpatient acute care and the emergency department. (DOCX) [file pone.0305381.s001.docx]

# Supplemental Table 1. Top 10 Reasons for Admission by International-Classification of Diseases (ICD) 10th Version Codes

| **Reason for Acute Hospitalization Visit by ICD-10 Codes** | **n (%)** |
| --- | --- |
| I50.0 | 533 (5.07) |
| A41.9 | 404 (3.84) |
| Z51.5 | 356 (3.38) |
| T87.47 | 311 (2.96) |
| E11.51 | 300 (2.85) |
| J18.9 | 246 (2.34) |
| I21.4 | 242 (2.30) |
| N39.0 | 238 (2.26) |
| E11.71 | 217 (2.06) |
| J44.0 | 183 (1.74) |
| **Reason for Emergency Department Visit by ICD-10 Codes** |  |
| N39.0 | 676 (3.44) |
| Z51.2 | 601 (3.06) |
| I50.0 | 591 (3.01) |
| L031.1 | 581 (2.96) |
| J18.9 | 468 (2.38) |
| Z48.0 | 464 (2.36) |
| T81.4 | 410 (2.09) |
| A41.9 | 396 (2.01) |
| R07.4 | 333 (1.69) |
| T81.3 | 260 (1.32) |
